# Qualitative Assay to Detect Dopamine Release by Ligand Action on Nicotinic Acetylcholine Receptors

**DOI:** 10.3390/toxins11120682

**Published:** 2019-11-20

**Authors:** Leanna A. Marquart, Matthew W. Turner, Owen M. McDougal

**Affiliations:** 1 Department of Chemistry and Biochemistry, Boise State University, Boise, ID 83725, USA; leannabrown@u.boisestate.edu; 2 Biomolecular Sciences PhD Program, Boise State University, Boise, ID 83725, USA; matthewturner1@u.boisestate.edu

**Keywords:** PC12 cells, alpha-conotoxin, nicotinic acetylcholine receptor, dopamine, luminescence assay

## Abstract

A pheochromocytoma of the rat adrenal medulla derived (a.k.a. PC12) cell-based assay for dopamine measurement by luminescence detection was customized for the qualitative evaluation of agonists and antagonists of nicotinic acetylcholine receptors (nAChRs). The assay mechanism begins with ligand binding to transmembrane nAChRs, altering ion flow into the cell and inducing dopamine release from the cell. Following release, dopamine is oxidized by monoamine oxidase generating hydrogen peroxide that catalyzes a chemiluminescence reaction involving luminol and horseradish peroxidase, thus producing a detectable response. Results are presented for the action of nAChR agonists (acetylcholine, nicotine, and cytisine), and antagonists (α-conotoxins (α-CTxs) MII, ImI, LvIA, and PeIA) that demonstrate a luminescence response correlating to the increase or decrease of dopamine release. A survey of cell growth and treatment conditions, including nerve growth factor, nicotine, ethanol, and temperature, led to optimal assay requirements to achieve maximal signal intensity and consistent response to ligand treatment. It was determined that PC12 cells treated with a combination of nerve growth factor and nicotine, and incubated at 37 °C, provided favorable results for a reduction in luminescence signal upon treatment of cells with α-CTxs. The PC12 assay is intended for use as a fast, efficient, and economic qualitative method to assess the bioactivity of molecules that act on nAChRs, in which testing of ligand–nAChR binding hypotheses and computational predictions can be validated. As a screening method for nAChR bioactivity, lead compounds can be assessed for their likelihood of exhibiting desired bioactivity prior to being subjected to more complex quantitative methods, such as electrophysiology or live animal studies.

## 1. Introduction

Nicotinic acetylcholine receptors (nAChRs) are pentameric transmembrane proteins present throughout the nervous system, brain, and muscle. Neuronal nAChRs are involved in critical brain functions, and have been implicated in a variety of neurological diseases and disorders [1,2,3,4,5]. Treatment options for neurological diseases, including Parkinson’s, Alzheimer’s, schizophrenia, and nicotine addiction, are limited by insufficient understanding of nAChR-mediated cell signaling in response to ligand binding [6,7]. This gap in knowledge exists due to a multitude of factors, which include the lack of nAChR structure data due to the difficulty in crystallizing these integral membrane proteins, the absence of robust heterologous non-recombinant expression systems [8], and the complexity and accessibility of experimental methods to study ion flow through nAChRs across cell membranes [9,10]. Current methods of identifying ligand binding paradigms with nAChR subtypes include computational docking and molecular dynamics simulations [11,12,13,14,15,16,17,18,19,20,21,22,23]; two-electrode voltage clamp electrophysiology with *Xenopus* oocytes that express the desired nAChR isoform [24]; patch clamping in cells over-expressing nAChRs, such as human embryonic kidney (HEK) and neuroblastoma cells [25]; model studies using acetylcholine binding proteins (AChBPs) [26,27,28,29]; radiolabeling of nAChR antagonists; investigations using mouse brain slices [30,31,32]; and in vivo trials with animals [33] or humans [34]. Cell-based assays using fluorescent indicators, such as the fluorometric imaging plate reader(FLIPR) fluorescent membrane potential assay or the Flou-4AM calcium indicator assay, are being used with increasing frequency to study nAChR activation [35]. Each of these strategies to study nAChR bioactivity are challenging to implement in their own unique ways. The use of computational docking and molecular dynamics simulation software programs to study the interaction of ligand binding to nAChRs has been significantly limited in the prediction of bioactivity, because binding affinity indirectly correlates with the physical dynamic changes associated with pore opening and closing of the membrane-bound channel [36]. Computational methods can be used to calculate the binding affinity for small molecule drugs by screening compound libraries, but correlating the binding affinity to bioactivity is inherently inaccurate, and fails further in the assessment of a compound as an agonist or antagonist, necessitating wet-lab validation. Studies utilizing AChBPs allow assessment of ligand binding, but not the ion flow across a membrane required to trigger signaling mechanisms within a cell that results in dopamine release, because the AChBP lacks a transmembrane component. Electrophysiology experiments require extensive expertise, highly specialized equipment, and significant investment of time to obtain results. Using electrophysiology to evaluate a wide range of compounds in order to test hypotheses about binding paradigms is impractical, either because the time and expense required to screen the compounds is so intensive, or because of the inaccessibility of high-throughput and automated methods [9,10,37,38]. Mouse brain studies involve probing slices of mammalian brain with fluorescently labeled conotoxin (CTx) to observe localized binding regions [32,39]. However, the resources required to work with mammalian tissue or living animals, including animal centers and oversight boards, can present prohibitive challenges for researchers at many institutions. There is a need to establish methods that bridge the gap between computational results and extensive experimentation, such that predicted compounds can be proven to demonstrate desired bioactivity prior to exhaustive and expensive laboratory evaluation. An accessible, time and cost efficient, and reliable PC12 cell method to qualitatively assess the ligand effect on nicotinic acetylcholine receptor-mediated control of dopamine release is presented. 

**Figure 1:**
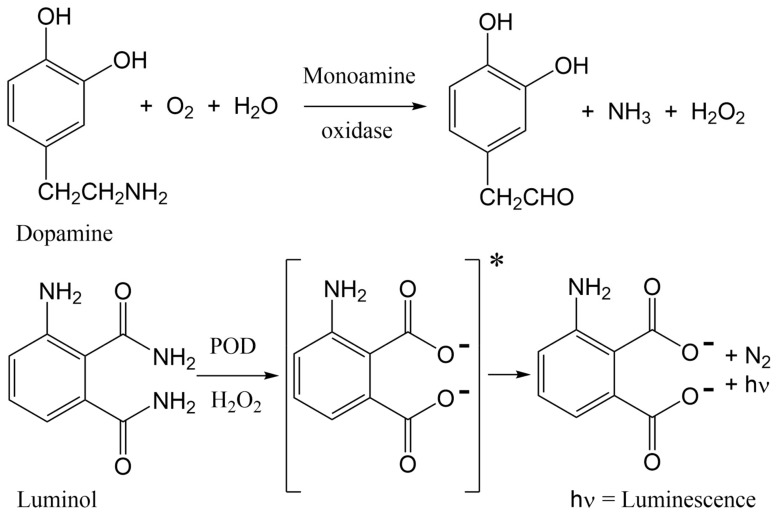
Chemical changes proposed to occur in the PC12 assay, from nAChR-induced dopamine release to luminescence detection. More dopamine release leads to a greater signal. Antagonists of nAChRs block dopamine release, diminishing signal.

The PC12 protocol reported in this study is an adaptation of a luminescence bioactivity assay that permitted measurement of dopamine release from rat PC12 cells, following activation by acetylcholine (ACh), bradykinin, muscarine, and ATP [40,41]. Acetylcholine binds to both nicotinic and muscarinic acetylcholine receptors (mAChRs), bradykinin activates bradykinin receptors B1 and B2, muscarine activates mAChRs, and ATP activates P2X and P2Y purinoreceptors. Acetylcholine stimulates only n/mAChRs to release dopamine, while the other agonists (bradykinin, muscarine, and ATP) operate by alternative signaling pathways to release neurotransmitters that contribute to the signal measured by luminescence detection. The original work by Shinohara et al. demonstrated that real-time dopamine release by PC12 cells, upon stimulation by acetylcholine, permitted luminescence detection in a dose-dependent manner, and enhancement of dopamine release was achieved by augmentation of assay conditions to include nerve growth factor (NGF). Shinohara et al. presented their assay as a useful tool to assess drugs that affect the nervous system. Building upon the precedent set by Shinohara et al., our lab has adapted the PC12 assay for assessment of ligands that specifically target nAChRs, as opposed to muscarinic or ATP receptors that induce dopamine release through alternate pathways. In the presence of agonists, including ACh, nicotine, or cytisine, nAChR channels will open to permit sodium ions into the cell. This influx of ions stimulates calcium ion influx, resulting in vesicle expulsion of dopamine from PC12 cells. When dopamine is released from the cell, monoamine oxidase (MAO) oxidizes dopamine to generate hydrogen peroxide, which reacts with luminol in the presence of horseradish peroxidase (POD) to generate chemiluminescence, which can be measured by absorbance (Figure 1).

In the present investigation, the PC12 assay protocol of Shinohara et al. was adapted for measurement of increased response following stimulation with ACh, nicotine, and cytisine [40,41]. Acetylcholine is a neurotransmitter that acts as the principal biological agonist of nAChRs. Nicotine and cytisine are alkaloids widely used in addiction studies involving nAChRs, because they both act as nAChR agonists due to their similar structural and pharmacological attributes. ACh, nicotine, and cytisine are all nonspecific agonists of most nAChR isoforms. EC_50_ and E_max_ values for agonist-stimulated ^86^Rb^+^ efflux in NGF-treated PC12 cells, as reported by Avila et al., are shown in Table 1 [42]. While it is now recognized that some nAChRs can bind G-proteins and induce neurotransmitter release through a G-protein coupling mechanism, as opposed to the more rapid ion translocation mechanism described above [43], the extracellular ligand effect on intracellular G-protein coupling of nAChRs remains unknown. However, numerous examples of associations between nAChRs with several types of G proteins have been demonstrated, which may contribute to crosstalk between nAChRs and G proteins [44,45,46]. 

**Figure 2:**
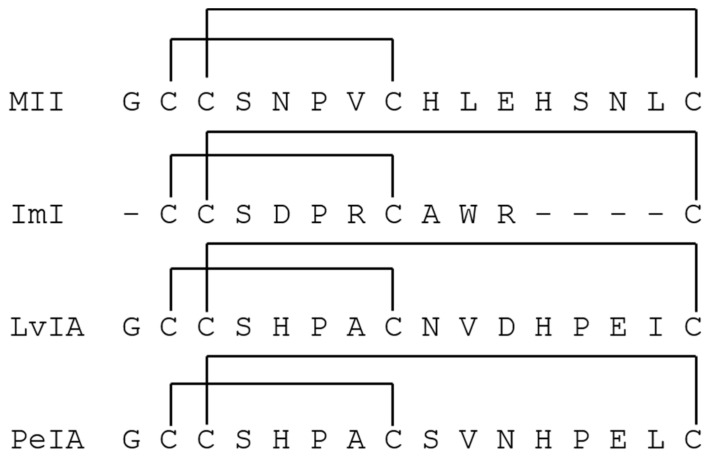
α-CTx peptide sequence and disulfide connectivity for rα3β2 nAChR antagonists evaluated in the PC12 assay.

α-Conotoxins (α-CTxs) are known to be highly potent and selective for nAChR isoforms, and have been used extensively by scientists seeking to understand ligand binding paradigms for nAChR subtypes [36]. The reason α-CTxs were selected as antagonists of nAChRs is because PC12 cells express mRNA coding for α3, α5, α7, β2, and β4 subunits but not α2, α4, α6, or β3 [47,48]. These subunits assemble into a variety of functional homo- and heteropentameric nAChRs, including predominantly α7, α3β4, and α3β2 [42,47,49]. Nery et al. used a strategy of non-specific nAChR stimulation followed by specific inhibition of the α7 nAChR isoform by methyllycaconitine citrate to inform the extent of activity for which the α7 isoform was responsible [47]. They concluded that PC12 cells induced to neuronal differentiation are a good model for studying α7 expression and activity in which various nAChR isoforms are expressed. This present study aimed to use the same strategy of non-specific nAChR stimulation followed by inhibition of nAChR isoforms to demonstrate the same conclusion regarding the rα3β2 nAChR isoform after optimizing growth conditions to favor its expression. Thus, to target ligand activity on rα3β2 nAChR subunits and demonstrate optimal assay conditions for its expression in PC12 cells, α-CTx ligands that selectively but differentially target the rα3β2 nAChR isoform were chosen. Suppression of rα3β2 nAChR-mediated cell signaling was evaluated using α-CTxs MII, ImI, LvIA, and PeIA (Figure 2). α-CTx MII is a 16-amino acid, 4/7 framework peptide, with primary sequence GCCSNPVCHLEHSNLC and disulfide bonds between C2–C8 and C3–C16, that selectively binds and inhibits rα3β2 nAChRs with an IC_50_ of 0.5 to 2.2 nM [50,51]. α-CTx MII has a >200-fold selectivity for rα3β2 as compared to either the α3β4 or α7 isoforms [50,52]. When used in the PC12 assay, suppression of luminescence response by α-CTx MII was interpreted as a measure of relative rα3β2 nAChR expression in the cell system. Of the nAChR subtypes expressed in the PC12 cells, α-CTxs ImI, LvIA, and PeIA were chosen due to their high selectivity for the rα7 (IC_50_ = 220 nM) [53], rα3β2 (IC_50_ = 8.7 nM) [19], and rα3β2 (IC_50_ = 19.2 nM) [54] nAChR subtypes, respectively. The IC_50_ values for ImI, LvIA, and PeIA with rα3β2 nAChR are >5 µM [53], 8.7 nM [19], and 19.2 nM [54], respectively. The IC_50_ values for ImI, LvIA, and PeIA with rα3β4 nAChR are >5µM [53], 148 nM [19], and 480 nM [54], respectively. This sampling of α-CTx antagonists was used to establish the reliability of achieving a qualitative luminescence response from ligand interaction with nAChRs that led to the excretion of dopamine. Each of these α-CTxs acts on nAChRs, but their effect can only be qualitatively assessed using this assay, due to their activity on multiple nAChR isoforms with varying affinity.

PC12 cell growth and ligand treatment parameters were evaluated for enhanced response to the rα3β2 nAChR isoform selective antagonist, α-CTx MII. PC12 assay results were assessed for influence by nerve growth factor, nicotine, ethanol, and temperature. Rogers et al. revealed that PC12 cells express the rα3β2 nAChR isoform among other receptor subtypes, and that NGF treatment provides growth conditions that favor expression of the β2 subunit [55]. In a complementary study, Shinohara et al. showed that NGF treatment enhanced luminescence response in the PC12 bioactivity assay, suggesting that NGF may increase nAChR expression [41]. Nerve growth factor-treated cells were evaluated in the presence of agonist and antagonist to determine whether NGF treatment would enhance the signal-to-noise ratio for the luminescence response. A separate investigation by Dohrman et al. demonstrated that addition of nicotine and ethanol to PC12 cells amplified nAChR expression [56]. Walsh et al. were able to enhance upregulation of rα3β2 nAChR expression, in HEK cells transfected with cDNA, upon long-term exposure to nicotine [57]. Cooper et al. observed that when TSA201 cells co-transfected with α4 and β2 cDNA were incubated at 30 °C instead of 37 °C, a 12-fold increase in α4β2 nAChR radioligand binding was observed with no changes in binding affinity, and a 5-fold increase in cell-surface receptors was achieved with no increase in total protein [58]. Avila et al. demonstrated through epibatidine binding and ^86^Rb^+^ efflux studies that NGF treatment predominantly increases expression of an α3β4 receptor subtype in PC12 cells, while nicotine treatment predominantly increases expression of an α3β2 receptor subtype, and that these two treatments combined have a supplemental effect on increasing nAChR expression [42]. However, radioligand binding and ^86^Rb^+^ efflux studies do not provide real-time observation of dopamine release through nAChR stimulation or suppression by ligands, such as the study by Shinohara et al., which the current study aimed to achieve.

Given the wealth of data associated with nAChR expression in PC12 cells reported in the literature, the PC12 cell assay conditions optimized in the present study were performed to achieve two objectives. The first objective was to obtain an optimal response for nAChR agonist and antagonist activity by alteration of the PC12 assay experimental parameters, including NGF, nicotine, ethanol, and temperature. The second objective was to establish optimal α-CTx suppression of dopamine release by inhibition of nAChRs in the context of the PC12 cell line expression system, as recorded by luminescence response.

## 2. Results

**Figure 3:**
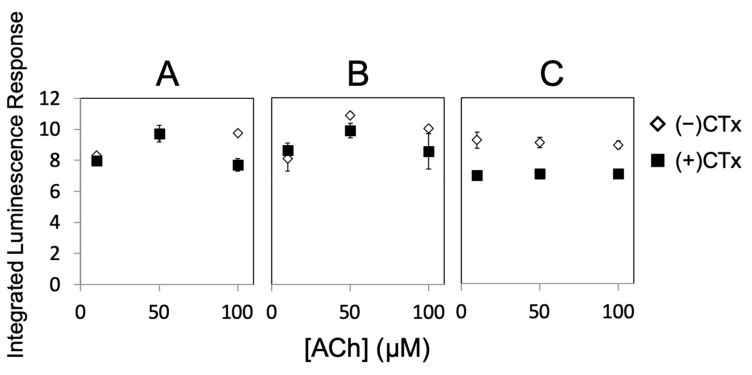
Integrated luminescence response versus ACh for (**A**) NGF(−), (**B**) NGF(+), and (**C**) NGF/nicotine. ACh response at 0 µM is not shown; the first point in each plot corresponds to the 10 µM ACh treatment to maximize visualization of data points and error bars.

Figure 3 (A–C) shows the luminescence response following stimulation by ACh for PC12 cells under standard conditions (3A), treated with NGF (3B), and a combination of NGF and nicotine (3C). Treatment conditions were sustained for 3 to 4 days. Stimulated response by ACh is indicated by black squares and the suppression of response in the presence of α-CTx MII is indicated by white diamonds. ACh and α-CTx MII were used as a representative agonist/antagonist pair for non-specific nAChR stimulation and selective nAChR suppression in order to provide the clearest sensitivity of the α-CTx effect on nAChR-induced dopamine release. 

Non-NGF-treated cells (Figure 3A) give a lower luminescence response than NGF-treated cells (Figure 3B), and addition of α-CTx MII leads to a decrease in luminescence response for both non-NGF and NGF-treated cells. Treatment with nicotine relatively decreases luminescence, but increases sensitivity to α-CTx MII (Figure 3C). An additional PC12 assay was performed for the combined treatment with NGF, nicotine, and ethanol, which increased the relative luminescence response but decreased sensitivity to α-CTx MII. Unfortunately, the variability in the luminescence response under these treatment conditions did not lead to a reliable result and data are not shown here.

**Figure 4:**
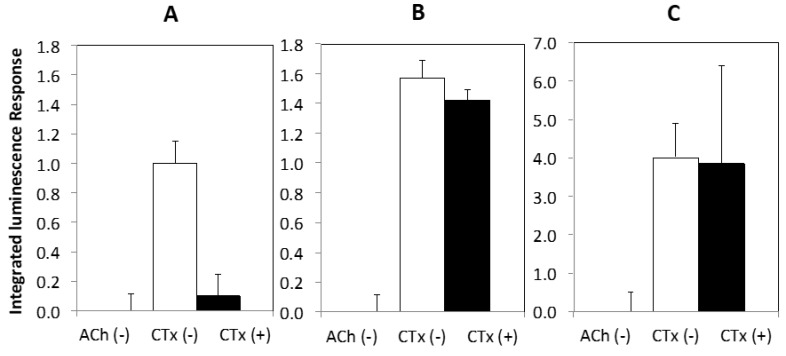
Luminescence response in the PC12 assay for normal cells incubated at 37 °C (**A**), and cells incubated at 30 °C for 1 h (**B**) and room temperature (21 °C) for 2 h (**C**) before detection. Incubating cells at a lower temperature before detection significantly increases response in a temperature-dependent manner but decreases sensitivity to α-CTx MII.

Effective NGF treatment was confirmed by cell morphology, in which NGF-treated cells contained neurite extensions indicative of neuron-like differentiation (Figure S1). According to the treatment study in Figure 3, chemical treatment with NGF and nicotine provided the most apparent non-specific stimulation of nAChRs by ACh and most obvious suppression of nAChR-induced dopamine release by α-CTx MII (Figure 3C). The optimal PC12 assay conditions consisting of NGF and nicotine were further evaluated for temperature dependence at 37, 30, and 21 °C for 1 to 2 h before detection of the luminescence response (Figure 4). 

Cells incubated at a lower temperature before luminescence recording showed a significantly increased response in a temperature-dependent manner but sensitivity to α-CTx MII treatment was appreciably diminished. The luminescence response for cells incubated at 37 °C was appreciably higher as compared to those incubated at lower temperatures.

**Figure 5:**
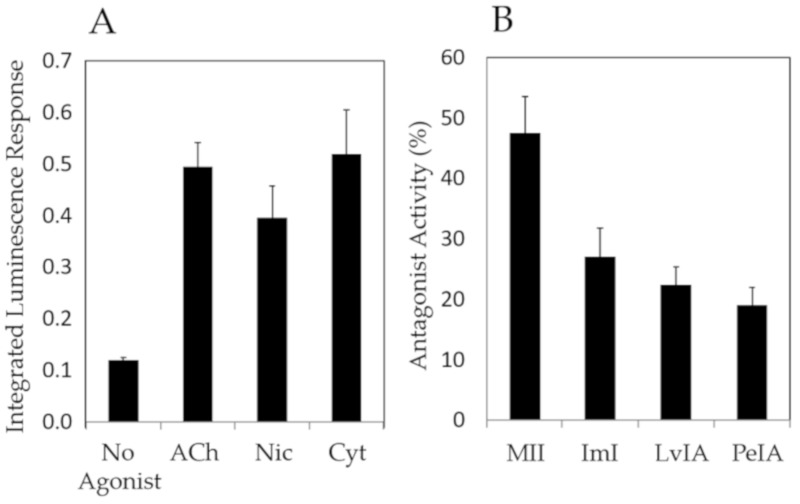
PC12 assay screen using (**A**) 50 µM nAChR agonists (acetylcholine, nicotine, cytisine), and (**B**) 10 µM nAChR antagonists (α-CTxs MII, ImI, LvIA, PeIA) upon stimulation with ACh, under the optimal growth conditions identified in Figure 3 and Figure 4 (treatment with NGF and nicotine, and incubation at 37 °C). The PC12 assay provided qualitative bioactivity assessment for each agonist and antagonist.

Finally, Figure 5 shows the results of a compound screen with select nAChR agonists (ACh, nicotine, cytisine) and antagonists (α-CTxs MII, ImI, LvIA, PeIA) upon ACh stimulation, using the optimal conditions identified as a result of the investigations in Figure 3 and Figure 4, which included chemical treatment with NGF and nicotine, and incubation at 37 °C.

Figure 5 shows that agonists amplified the luminescence response and antagonists suppressed the luminescence response, demonstrating that this PC12 assay may be used for qualitative assessment of ligands that bind nAChRs.

## 3. Discussion

Decreased luminescence response upon treatment with α-CTx MII indicates that α-CTx MII inhibition of nAChR-mediated release of dopamine in the PC12 cell assay can be observed and measured (Figure 3). Sensitivity to α-CTx MII antagonism was used as a measure of the relative expression of the rα3β2 nAChR isoform, because α-CTx MII is a highly selective and potent inhibitor of rα3β2 nAChR isoforms, while other nAChR isoforms expressed in PC12 cells are inhibited by α-CTx MII to a much lesser degree. Additionally, the increased luminescence response with NGF treatment (Figure 3B) suggests that NGF increases the population of functional nAChRs on the surface of the PC12 cells. Increasing the concentration of nAChRs expressed is favorable for increasing the sensitivity of the PC12 assay. An increase in luminescence upon NGF treatment is consistent with the findings of Shinohara et al. [41].

Sensitivity to α-CTx MII treatment increased when both nicotine and NGF were used, suggesting that rα3β2 nAChR isoform expression is greater under this combined treatment condition (Figure 3C). This result is consistent with the findings of Walsh et al., who reported higher rα3β2 nAChR expression upon long-term exposure to nicotine in HEK cells transfected with cDNA [57]. Slightly decreased luminescence response upon 3 to 4 days of treatment with nicotine in combination with NGF can be interpreted as a decrease in the number of functional cell-surface nAChRs as a result of nicotine treatment, as compared to solely NGF-treated cells (Figure 3C). These results correlate well with the findings of Dohrman et al., who demonstrated that chronic nicotine induces overall upregulation of nAChRs in PC12 cells [56]. To detect the presence of nAChRs on the cell surface, Dohrman et al. used ^125^I-labelled epibatidine, which binds more potently to α3β2, α3β4, and α4β2 nAChR subtypes than acetylcholine. Additionally, epibatidine binding requires incubation at room temperature for 1 h, which our results have shown drastically increases response (Figure 4), and suggests a subtype-nonspecific increase in nAChR cell-surface expression. It is possible the observation by Dohrman et al. of overall upregulation of nAChRs in PC12 cells does not specifically reflect physiological expression of functional cell-surface nAChRs at 37 °C. An increase in cell-surface expression upon incubation at lower temperatures is consistent with the findings of Cooper et al., who observed that when TSA201 cells co-transfected with α4 and β2 cDNA were incubated at 30 °C instead of 37 °C, there was a five-fold increase in cell-surface receptors with no increase in total protein [58]. Further support for this hypothesis is provided by Lukas et al., who found that chronic (3–72 h) nicotine treatment led to nearly a complete loss of the functional nAChR response in PC12 cells by nicotine and the cholinergic agonist carbamylcholine [59]. It is possible with the combination of the PC12 cell line, treatment exposure time, and dosage used, that functional inactivation is seen instead of functional upregulation.

For normal cells treated with nicotine for 3 to 4 days and incubated at 37 °C (Figure 4A), a >50% suppression of response to 50 µM ACh by α-CTx MII was observed. Cells incubated at lower temperatures (Figure 4B,C) before luminescence detection showed a significantly increased response compared to cells at 37 °C. This is consistent with the findings of Cooper et al., who observed that when TSA201 cells co-transfected with α4 and β2 cDNA were incubated at 30 °C instead of 37 °C, there was a five-fold increase in cell-surface receptors with no increase in total protein. While PC12 cells did not survive at 30 °C for 24 h or more, incubating at 30 and 21 °C for only 1 to 2 h before detection showed a significant and temperature-dependent increase in luminescence response; however, sensitivity to α-CTx MII was essentially eliminated. Decreased sensitivity to α-CTx MII may suggest a subtype-nonspecific increase in nAChR cell-surface expression.

In summary, combined treatment with NGF and nicotine, and incubation at 37 °C provides the greatest level of sensitivity to α-CTx MII. Despite extensive repetition to obtain the results presented, it appears the PC12 assay may be limited to a comparison of results within the same well plate. It is hypothesized that the rapid growth of PC12 cells changes their condition too dynamically from subculture to subculture to permit comparison of luminescence intensity directly across well plates. Inconsistent expression from cell to cell, high background noise, and dynamic changes between subcultures can complicate results and cause absolute luminescence intensities and integrated luminescence response to differ widely across well plates, explaining the variability in absolute luminescence measurements in Figure 3, Figure 4 and Figure 5. Regardless of these limitations, the PC12 assay conditions presented here can be applied to qualitative characterization of bioactivity of lead compounds with affinity for nAChRs. 

The PC12 assay was examined for use as a qualitative bioactivity test for a range of known nAChR agonists (acetylcholine, nicotine, and cytisine) and antagonists (α-CTxs MII, ImI, LvIA, PeIA) (Figure 5). The PC12 assay accurately provided signal amplification in the presence of nAChR agonists (Figure 5A), and signal suppression upon treatment with nAChR antagonists (Figure 5B). These results further reflect relative potency between α-CTx MII and other less potent antagonists, with α-CTx MII showing the highest antagonistic activity. The responses obtained by this collection of α-CTx antagonists indicate that nAChR-mediated channel closing may be assessed qualitatively. α-CTx MII is known to potently and selectively suppresses luminescence response generated by rα3β2 nAChR isoform, and was interpreted in this study as a measure of relative rα3β2 nAChR expression in the cell system. α-CTx MII achieved a 50% suppression of the luminescence response, suggesting a relatively high level of expression of functional rα3β2 nAChR subtypes in the cell system. The levels of suppression observed in Figure 5 by α-CTxs MII, ImI, LvIA, and PeIA reflect their relative specificities for rα3β2, rα7, rα3β2 > rα3β4, and rα3β2 > rα3β4, respectively, and suggest that rα3β2, rα7, and rα3β4 nAChRs are present to a high detectable degree in PC12 cells, consistent with previous literature [42,47,49].

This study presents an accessible, time and cost efficient, and reliable luminescence-based method for the detection of nAChR bioactivity, and demonstrates decreased response upon addition of α-CTx antagonist. As a result of the alterations made to the PC12 protocol (see Supplementary Materials), this assay can be performed at any institution with a microplate reader that can detect luminescence. Simplifications to the protocol, such as a reduction in the amount of required enzymes and eliminating the need for trypsinization, significantly reduce material costs. This study provides a simple method for measurement of the nAChR bioactivity response that can be quickly and easily performed. Because PC12 cells grow quickly, the time from cryopreservation recovery to assay can be achieved within one week with 3 to 4 days of treatment. One response curve can be obtained within minutes, enabling evaluation of an entire 96-well plate within an hour. Optimal growth conditions for expression of rα3β2 nAChRs were measured by the degree of sensitivity to α-CTx MII suppression. According to the results of this study, combined treatment with NGF and nicotine, and incubation at 37 °C provides the greatest level of sensitivity to α-CTx MII. This adapted PC12 assay can be applied to qualitative characterization of bioactivity of lead compounds with nAChRs, as demonstrated by the compound screen performed here under optimized growth conditions with non-specific agonists and antagonists of nAChRs. 

Shinohara et al. permitted real-time detection of dopamine release upon stimulation of nAChR and bradykinin receptor agonists in PC12 cells without NGF treatment, as well as upon stimulation of mAChR, ATP receptor, and bradykinin receptor agonists in PC12 cells treated with NGF. The present investigation improves upon Shinohara’s original assay by exploring and identifying optimal growth conditions for nAChR expression, and the use of selective α-CTxs to assess nAChR isoforms responsible for assay response upon stimulation with ACh, demonstrating that nAChR expression can be manipulated by chemical treatment and growth conditions in order to qualitatively characterize the real-time ligand effect on nAChR-induced dopamine release. While we demonstrated that this assay provides accurate bioactivity validation, it is apparent that the assay’s sensitivity is limited by a response resulting from the presence of non-rα3β2-nAChR subtypes. Additionally, inconsistent expression from cell to cell, and high background noise can complicate results. Possible reasons for such background noise in the response include inherent base-level activity of predominantly expressed nAChRs, such as rα7, rα3β4, and rα3β2, as well as G-protein coupled activation of nAChRs as opposed to ion translocation [43]. While this mechanism of nAChR activation is now recognized in the literature, it is uncertain how the ligands evaluated here, which are responsible for affecting nAChR activity through ion translocation, might affect nAChR coupling to G-proteins. Thus, it is expected that the response obtained from G-coupled activation of nAChRs is corrected with the negative controls used in this study. Future research directions include the adaptation of this assay coupled to overexpression of individual nAChR isoforms, or knockout or knockdown of particular nAChR subunits, resulting in an assay in which the pharmacology of a single nAChR subtype may be evaluated. This assay serves as a foundation for the design of engineered PC12 cell lines expressing specific human nAChR isoforms to enable the study of lead compounds for the treatment of neurological diseases in which non-rα3β2-nAChR isoforms are implicated (e.g., Parkinson’s, schizophrenia, and Alzheimer’s). The ease and simplicity with which PC12 cells can be cultured and manipulated is widely reported [60], and makes PC12 cells an ideal cell line for the investigation of ligands that act on nAChRs.

## 4. Conclusions

A simple method for the measurement of ligand-induced bioactivity on nAChRs that can be quickly and easily performed was presented. Because PC12 cells grow quickly, the time from cryopreservation recovery to assay can be achieved within one week with 3 to 4 days of treatment. Assay response curves can be obtained within minutes, enabling evaluation of an entire 96-well plate within an hour. Optimal growth conditions for expression of rα3β2 nAChRs were measured by the degree of sensitivity to α-CTx MII suppression. According to the results of this study, combined treatment with NGF and nicotine, and incubation at 37 °C provides the greatest level of sensitivity to α-CTx MII. This adapted PC12 assay can be applied to qualitative characterization of the bioactivity of lead compounds with nAChRs, as demonstrated by the compound screen performed here under optimized growth conditions with non-specific agonists and antagonists of nAChR. 

## 5. Materials and Methods 

The materials and methods for this PC12 assay represent an adaptation and modification of the foundational work originally reported by Shinohara et al. [40,41]. Key variations to their reported method are discussed in greater detail in the Supplementary Materials.

### 5.1. Materials

Assays were performed using a Biotek Synergy H1 microplate reader (Winooski, VT, USA). All chemicals were purchased at the highest purity available (>95%) from Fisher Scientific, except for synthetic α-CTxs, which were purchased from CS Bio Inc. (Menlo, CA, USA). ATCC® CRL1721™ PC12 cells were provided by the Biomolecular Research Center at Boise State University.

### 5.2. Methods

*Cell Culture:* PC12 cells were recovered from cryopreservation, seeded, and cultured into laminin-coated flasks according to Shinohara et al. with the exception that cells were not trypsinized to detach them from laminin [40]. Instead, cells were triturated from the bottom of the flask. Cells were initially grown in a T-75 flask until dense enough to plate onto either a laminin-coated flat-bottom 96-well plate at 10,000 cells per well, or 48-well plate at 30,000 cells per well, taking care to sufficiently disperse cell clumps when triturating. Plating at a sufficiently low density and ensuring the distribution of cells to evenly cover each well bottom was crucial to obtaining consistent assay response. After subculturing into a well plate, cells were allowed to equilibrate for 24 h before exchanging complete growth media for treated starvation media (30 ng/mL NGF 2.5S, 1 µM nicotine, 100 mM ethanol, in the desired combinations of treatment) and treating for 3 to 4 days before performing the assay, switching out fresh starvation media daily. Treatment concentrations of nicotine and ethanol were based on those used by Dohrman et al. [52]. Well plates were sealed with Parafilm during treatment to prevent evaporation of ethanol from treatment media.

*Cell Assay:* Cells were washed with Locke’s solution (pH 7.4) before introducing assay solution. An assessment of modified Hank’s balanced salt solution was performed but results were unfavorable (Figure S2). Assay solution included 10 µM α-CTx MII, 0.8 ng/mL horseradish peroxidase (POD), 25 ng/mL monoamine oxidase (MAO), and 50 µM luminol to quench any dissolved oxygen in the cell environment and the assay solution. Cells were allowed to sit for 5 to 10 min to allow equilibration, before adding 2 mM luminol and 50 µM agonist (acetylcholine, cytisine, or nicotine) immediately before detection, for a total well volume of 100 µL. (-)MAO and (-)cells were used as negative controls, replacing the volume with Locke’s solution. Assays were performed at 37 °C, detecting luminescence for 1 to 2 min per well or until the response fully diminished to negative control levels in order to obtain a complete curve. Once luminescence response curves were obtained, curves with abnormal shapes indicating obvious faults in detection caused by anomalies, such as air bubbles, were excluded. Curves were then integrated and the area under the curve was used to build dosage curves for luminescence response versus concentration of agonist. To evaluate the influence of temperature on the assay results for a single well plate to avoid the introduction of unnecessary variation across well plates, cells were prepared as above, but after detecting the response for a portion of the wells at 37 °C, the remaining wells were incubated at either 30 °C or room temperature (21 °C) for 1 to 2 h. The assay was then resumed after switching media with assay solution incubated at the corresponding lower temperature into the remaining wells for detection.

## Figures and Tables

**Table 1:** EC_50_ and E_max_ values for agonist-stimulated ^86^Rb^+^ efflux in NGF-treated PC12 cells for acetylcholine, nicotine, and cytisine. EC_50_ and E_max_ values are in units of μM [42].

Agonist	EC_50_	E_max_
Acetylcholine	118 ± 23	307 ± 24
Nicotine	33 ± 1.5	232 ± 23
Cytisine	42 ± 8.6	256 ± 21

## Supplementary Materials

The following are available online at https://www.mdpi.com/2072-6651/11/12/682/s1, Figure S1: Light microscopy images of PC12 cell morphology, Figure S2: Visual state of PC12 cells pre- and post-assay.
