# Supplementary material for: Qualitative Assay to Detect Dopamine Release by Ligand Action on Nicotinic Acetylcholine Receptors
Source: Toxins (Basel). 2019 Nov 20;11(12):682. doi: 10.3390/toxins11120682 (PMC6949981; doi:10.3390/toxins11120682)
Supplement: Supplementary file 1 [file toxins-11-00682-s001.pdf]

Supplementary Materials: Qualitative Assay to Detect Dopamine Release by Ligand Action on Nicotinic Acetylcholine Receptors

Leanna A. Marquart, Matthew W. Turner and Owen M. McDougal

Method Optimization and Troubleshooting

Effective NGF treatment was confirmed by cell morphology, in which NGF-treated cells contained neurite extensions indicative of neuron-like differentiation (Figure S1).

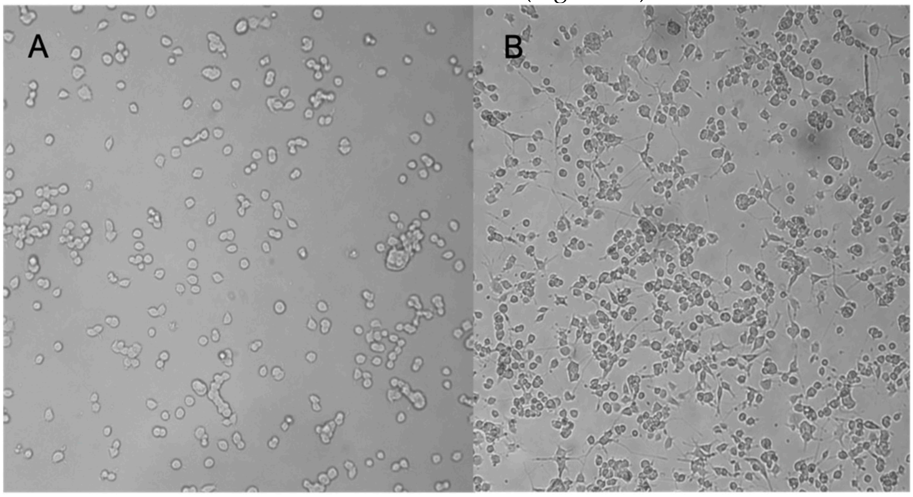

Figure S1. Light microscopy images of PC12 cell morphology. Non-NGF-treated cells (**A**) remain round, while NGF-treated cells (**B**) show long, reaching dendrites at 3 days. No visual difference was observed in cell morphology for treatments including nicotine and/or ethanol in addition to NGF.

The trypsinization step used by Shinohara et al. was removed, not only to simplify growth procedures but also because it has been shown that trypsinization is damaging to cell surface proteins [1]. In order to prolong the life of the PC12 cells and maximize assay response, cells were instead triturated from the bottom of the flask to detach them for subculturing. It was observed that without trypsinization, cells responded well to ACh over a longer period of time, 2 to 3 weeks from cryopreservation recovery. The results presented in this study are from cells that were cultured for at least 2 weeks. Extensive optimization was necessary to adapt the Shinohara et al. method to the 96-well plate α-CTx MII-dependent rα3β2 nAChR isoform specific response system, which constituted the objective for the current study. Adaption of Shinohara's method was designed such that reliable results could be obtained for studies involving rα3β2 nAChR isoform-specific antagonists, as represented by α-CTx MII, as well as adapting the protocol for accessibility, and time and cost efficiency. Initial attempts to recreate the results of Shinohara et al. proved challenging due to a lack of access to an autoinjector for instantaneous detection of ACh response. To overcome this deficiency, it was necessary to decrease [POD] and [MAO] in order to slow down the reaction and increase [luminol] so that the luminescence response could be delayed beyond the time required for pipette addition of ACh to the beginning of detection by the microplate reader. Slowing the reaction resulted in a broader luminescence detection peak from 30 to 60 s, rather than the rapid, sharp peak over 2 to 3 s observed by Shinohara. Thus, it was not practical to measure the maximum luminescence intensity for each peak when interpreting data, as was reported by Shinohara. Instead, the area under the curve was measured for a more accurate, reliable interpretation of luminescence response.

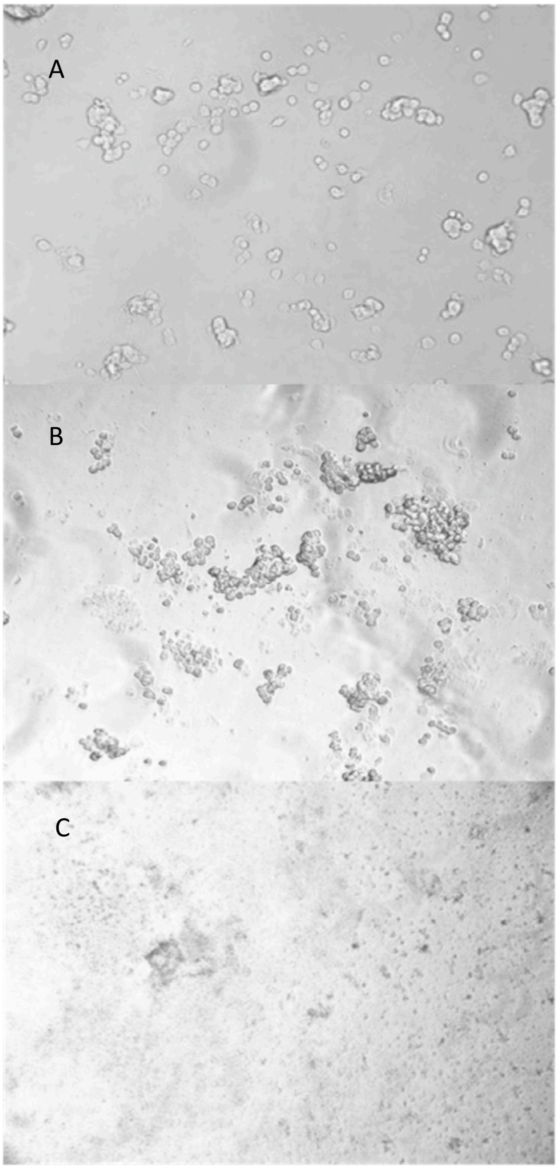

Figure S2. Visual state of PC12 cells pre-assay (**A**) and post-assay after performing the assay with both Locke's buffer (**B**) and HBSS (**C**). Cells post-assay using HBSS appeared severely lysed and damaged.

It is important to note that one limitation to the PC12 assay is that results must be compared within the same well plate, since the rapid growth of PC12 cells changes their condition too dynamically from subculture to subculture to permit comparison of luminescence intensity directly across well plates. The goal for the treatment study in Figure 3 was to compare four different PC12 growth conditions for luminescent response upon treatment with and without α-CTx MII and optimize the concentration of ACh for maximum sensitivity. Thus, to be able to compare all four treatment conditions (NGF, nicotine, ethanol, and combined nicotine and ethanol) together over a range of ACh doses for both +/- α-CTx MII with multiple negative controls, n = 2 was allowed for a full 96-well plate. The occurrence of faulty wells (for example, caused by anomalies, such as air bubbles or uneven conditions, at the edges of the well plate) reduced some data points to n = 1. Considering a cellular-level response was being detected, further reduction of the growth surface area by using a 192- or 384- well plate was expected to introduce problems with sensitivity given the current level of progress toward optimization of nAChR expression. For an

experiment in which the goal of the assay was to qualitatively screen a range of compounds, a simpler scheme with one dose of 50 μM ACh would be sufficient, allowing for more statistically complete data. For example, a more statistically complete result is demonstrated in the temperature study shown in Figure 5, for which an n = 4 was used.

It was found that a better zero response for 0 μM ACh was obtained when a small amount of luminol was added to the assay solution before detection. Luminol addition was used to quench residual oxygen dissolved in the assay solution or cell environment that was capable of breaking down the luminol and cause a luminescence response not relevant to dopamine release. The luminol addition provided a more level base response across all wells. It was found that the cells consume this small amount of luminol (50 μM) very quickly over the 5 to 10 min equilibration time even without activation by ACh, necessitating the addition of a larger dose of luminol along with the ACh before detection. In an attempt to maximize the sensitivity of the assay, the luminol concentration was arbitrarily spiked to 5 mM, for which a consistent assay response was not achieved. It was then considered that the 1 M NaOH necessarily used to dissolve the luminol stipulated a maximum luminol concentration allowable before exceeding Locke's buffer capacity. Thus, while maximum luminescence response was desired, the maximum allowable [luminol] was limited by the buffer capacity.

Subsequent to the release of Shinohara et al.'s initial study in 2008, Locke's buffer was replaced with modified Hank's balanced salt solution (HBSS) in their 2011 study [2,3]. In step with their decision, when simple HBSS was attempted in this study, interpretable results were drastically eliminated. Both buffers have a comparable osmolality of 270 to 305 mOsm/kg, and the greatest difference between Locke's buffer and simple HBSS is the absence of Mg^{2+} in Locke's buffer. After multiple unsuccessful attempts to recreate assay results with HBSS, it was discovered that the state of the cells post-assay under a microscope appeared surprisingly damaged and most cells were lysed. Figure S2 shows the state of the cells pre- and post-assay when using both Locke's buffer and HBSS. It is presumed that the modified HBSS used in Shinohara et al.'s 2008 study was Mg-free HBSS [2]. This conclusion is consistent with the literature precedent describing the depolarization of neuronal cells by Mg^{2+} [4].

References

1. Huang, H.L.; Hsing, H.W.; Lai, T.C.; Chen, Y.W.; Lee, T.R.; Chan, H.T.; Lyu, P.C.; Wu, C.L.; Lu, Y.C.; Lin, S.T., et al. Trypsin-induced proteome alteration during cell subculture in mammalian cells. *J. Biomed.Sci.* **2010**, *17*, 10, doi:10.1186/1423-0127-17-36.
2. Shinohara, H.; Wang, F.F.; Hossain, S.M.Z. A convenient, high-throughput method for enzyme-luminescence detection of dopamine released from PC12 cells. *Nat. Protoc.* **2008**, *3*, 1639–1644, doi:10.1038/nprot.2008.158.
3. Mir, T.A.; Shinohara, H.; Shimizu, Y. Enzyme-luminescence method: Tool for evaluation of neuronal differentiation based on real-time monitoring of dopamine release response from PC12 cells. *Anal. Methods* **2011**, *3*, 837–841, doi:10.1039/c0ay00769b.
4. Dribben, W.H.; Eisenman, L.N.; Mennerick, S. Magnesium induces neuronal apoptosis by suppressing excitability. *Cell Death & Disease* **2010**, *1*, 9, doi:10.1038/cddis.2010.39.
